# Self-reported Use of Prescribed Buprenorphine Among US Adults With Nonmedical Use of Prescription Opioids Motivated by Pain

**DOI:** 10.1001/jamanetworkopen.2022.41670

**Published:** 2022-11-11

**Authors:** Michele J. Buonora, Minhee L. Sung, Caroline G. Falker, Anne C. Black, William C. Becker

**Affiliations:** 1 National Clinician Scholars Program, Yale School of Medicine, New Haven, Connecticut; 2 Department of Internal Medicine & Program in Addiction Medicine, Yale School of Medicine, New Haven, Connecticut; 3 General Internal Medicine, Veterans Affairs Connecticut Healthcare System, West Haven; 4 Pain Research, Informatics, Multimorbidities & Education Center of Innovation, Veterans Affairs Connecticut Healthcare System, West Haven, Connecticut

## Abstract

This cross-sectional study of US adults examines the prevalence of and characteristics associated with prescribed buprenorphine use among US adults with pain-motivated nonmedical use of prescription opioids.

## Introduction

Persons engaging in nonmedical use of prescription opioids (NMUPO) because of pain are at risk for life-threatening consequences, including opioid use disorder (OUD) and overdose.^1,2^ Buprenorphine, a partial opioid agonist and effective treatment for OUD and pain, has fewer adverse effects than full agonists, including lower risk of overdose, and may be effective for persons who engage in NMUPO because of pain, representing a clinical spectrum from sporadic NMUPO to physiologic dependence to OUD.^3^ We sought to estimate the US prevalence of pain-motivated NMUPO and the proportion of individuals who self-report use of prescribed buprenorphine. We also identified clinical and health care use characteristics of the subpopulation not using prescribed buprenorphine to identify opportunities for overdose prevention.

## Methods

We conducted a cross-sectional study, which was determined to be exempt from review by the Yale University Institutional Review Board, of the 2018 and 2019 National Survey on Drug Use and Health (NSDUH), a nationally representative survey sponsored by the Substance Abuse and Mental Health Services Administration. We followed the STROBE reporting guideline. Data were analyzed with Stata/SE software, version 17.0 (StataCorp LLC).

We included all NSDUH respondents 18 years or older who reported (1) past-year NMUPO, defined as “use of prescription pain relievers in any way not directed by a doctor, including use without a prescription of one’s own medication; use in greater amounts, more often, or longer than told to take a drug”^4^ and (2) pain as a motivating factor for use (NSDUH question details in the eTable in the Supplement). Respondents receiving nonbuprenorphine treatment for OUD were excluded.

We compared the odds of past-year self-reported use of prescribed buprenorphine across variables—including self-reported race and ethnicity—associated with pain, OUD, and access to health care. We included data on race and ethnicity because prior work^5,6^ has established that racial and ethnic disparities exist in access to treatment for OUD, specifically buprenorphine. We conducted bivariate analyses, using χ^2^ and 2-tailed, unpaired *t* tests, and multivariable logistic regression. We calculated means and proportions of characteristics of the subpopulation not using prescribed buprenorphine, accounting for survey weighting for nationally representative estimates.

## Results

The US adult prevalence of NMUPO was 3.7%; 71.9% reported pain-motivated use, representing 6.6 million people. Within this cohort, the prevalence of self-reported use of prescribed buprenorphine was 6.8%. Factors associated with lower odds of using prescribed buprenorphine included Black race, part-time employment, and no insurance. Factors associated with higher odds of using prescribed buprenorphine included OUD diagnosis, tobacco use disorder, income at or below the federal poverty threshold, and unemployment (Table 1). Among the subpopulation not using prescribed buprenorphine, 14.1% were hospitalized in the past year, with a mean (SD) of 0.94 (0.06) emergency department visits and 4.1 (0.14) physician visits. A total of 40.3% reported the misused opioid was sourced directly from a physician (Table 2).

**Table 1.** Odds of Self-reported Use of Prescribed Buprenorphine Among US Adults With Pain-Motivated NMUPO

Person-level characteristic	Unweighted No./weighted No. (weighted %)		aOR (95% CI)^a^
	Persons with pain-motivated NMUPO (n = 2586/6 647 246)	Use of prescribed buprenorphine (n = 148/439 957)	
Demographic			
Age, y			
18-25	894/1 091 478 (16.4)	34/33 129 (7.5)	1 [Reference]
26-34	677/1 581 380 (23.8)	52/125 432 (28.5)	3.04 (1.40-6.66)
35-49	707/1 962 267 (29.5)	45/122 792 (27.9)	3.15 (1.40-7.11)
50-64	224/1 401 904 (21.1)	15/128 775 (29.3)	5.25 (1.75-15.80)
≥65	84/610 217 (9.2)	2/29 785 (6.8)	0.77 (0.06-10.20)
Sex			
Female	1342/3 211 949 (48.3)	78/215 491 (49.0)	0.93 (0.56-1.54)
Male	1244/3 435 297 (51.7)	70/224 466 (51.0)	1 [Reference]
Race and ethnicity			
Asian	53/169 505 (2.6)	2/12 495 (2.8)	5.17 (0.96-27.80)
Hispanic or Latino	460/1 078 848 (16.2)	21/104 974 (23.9)	2.11 (0.91-4.88)
Native American/Alaskan native	52/51 184 (0.8)	1/2244 (0.5)	0.57 (0.07-4.78)
Native Hawaiian or Pacific Islander	18/40 548 (0.6)	2/748 (0.2)	0.48 (0.06-3.88)
Non-Hispanic Black	291/731 862 (11.0)	8/19 226 (4.4)	0.42 (0.18-0.98)
Non-Hispanic White	1597/4 413 771 (66.4)	108/277 745 (63.1)	1 [Reference]
>1 Race	115/162 193 (2.4)	6/22 482 (5.1)	1.63 (0.40-6.61)
Clinical			
Opioid use disorder diagnosis^b^	343/1 021 682 (15.4)	68/260 675 (59.3)	6.34 (3.11-12.91)
Methadone prescription in past year	59/144 245 (2.2)	18/38 628 (8.8)	3.54 (0.95-13.17)
Source of opioid^c^			
1 Physician	882/2 553 872 (38.4)	54/182 494 (41.5)	1 [Reference]
>1 Physician	40/113 668 (1.7)	5/15 398 (3.5)	1.07 (0.25-4.63)
Stole from physician’s office	19/61 155 (0.9)	2/3828 (0.9)	0.46 (0.03-8.18)
From friend or relative for free	1014/2 573 814 (38.7)	32/89 179 (20.3)	0.69 (0.34-1.38)
Bought from friend or relative	214/547 068 (8.3)	27/95 999 (21.8)	2.33 (1.17-4.66)
Took from friend or relative without permission	79/194 764 (2.9)	3/9063 (2.1)	0.72 (0.15-3.39)
Bought from drug dealer	127/277 855 (4.2)	19/43 996 (10.0)	1.44 (0.34-6.06)
Some other way	112/325 050 (4.9)	1/0 (0.0)	0.001 (0.0001-0.01)
No. of medical comorbidities			
0	1672/3 860 056 (58.1)	84/275 941 (62.7)	1 [Reference]
1-2	854/2 548 554 (38.3)	58/141 930 (32.3)	0.54 (0.25-1.18)
≥3	60/238 636 (3.6)	6/22 130 (5.0)	0.23 (0.04-1.21)
Substance use comorbidities			
Cocaine use disorder^b^	94/198 753 (3.0)	18/44 876 (10.2)	1.69 (0.54-5.24)
Alcohol use disorder^b^	519/1 220 434 (18.4)	30/93 139 (21.2)	0.81 (0.42-1.60)
Tobacco use disorder^d^	702/1 848 599 (27.8)	82/220 022 (50.0)	1.86 (1.06-3.25)
Health care use			
No. of ED visits in past year			
0	1439/3 759 018 (56.6)	60/197 497 (44.9)	1 [Reference]
1-3	903/2 297 953 (34.6)	69/195 429 (44.4)	1.33 (0.69-2.58)
4-6	131/336 351 (5.1)	10/32 249 (7.3)	0.86 (0.24-3.08)
≥7	113/253 925 (3.8)	9/14 783 (3.4)	1.00 (0.32-3.13)
Any hospitalization in past year	2229/943 909 (14.2)	26/60 098 (13.7)	0.41 (0.16-1.10)
No. of physician visits in past year			
0	519/1 106 102 (16.6)	30/82 140 (18.7)	1 [Reference]
1-2	842/2 143 737 (32.3)	34/106 470 (24.2)	0.71 (0.34-1.48)
3-4	419/1 225 752 (18.4)	20/58 866 (13.4)	0.80 (0.34-1.89)
≥5	806/2 170 991 (32.7)	64/192 525 (43.8)	1.63 (0.69-3.86)
Socioeconomic			
Income to poverty level			
More than twice the federal poverty threshold	1359/3 806 213 (57.3)	58/157 857 (35.9)	1 [Reference]
Up to twice the federal poverty threshold	605/1 493 636 (22.5)	32/97 978 (22.3)	1.36 (0.61-3.06)
At or below the federal poverty threshold	595/1 347 397 (20.3)	58/184 078 (41.8)	3.30 (1.41-7.73)
Population density			
Large metropolitan	1145/3 531 017 (53.1)	59/206 296 (46.9)	1 [Reference]
Small metropolitan	973/2 199 574 (33.1)	61/170 219 (38.7)	1.51 (0.76-3.01)
Nonmetropolitan	468/916 655 (13.8)	28/63 442 (14.4)	0.85 (0.37-1.92)
Employment status			
Full time	1315/3 374 142 (50.8)	54/158 692 (36.1)	1 [Reference]
Part time	385/841 541 (12.7)	8/13 243 (3.0)	0.33 (0.14-0.74)
Unemployed	254/480 596 (7.2)	31/72 417 (16.5)	2.03 (1.04-3.97)
Other	632/1 950 967 (29.4)	55/195 561 (44.5)	1.35 (0.61-2.94)
Insurance type			
Private insurance	1166/2 891 552 (43.5)	40/113 861 (25.9)	1 [Reference]
Military or VA insurance	69/142 916 (2.2)	3/9811 (2.2)	0.95 (0.13-6.79)
Dual Medicare-Medicaid	80/287 161 (4.3)	6/19 138 (4.4)	0.22 (0.04-1.30)
Medicare	103/615 535 (9.3)	9/52 179 (11.9)	2.49 (0.46-13.40)
Medicaid	661/1 364 015 (20.5)	60/154 029 (35.0)	0.71 (0.31-1.60)
No insurance	438/1 163 933 (17.5)	27/73 781 (16.8)	0.48 (0.20-0.99)
Other	69/181 470 (2.7)	3/17 114 (3.9)	0.41 (0.05-3.31)

Abbreviations: aOR, adjusted odds ratio; ED, emergency department; NMUPO, nonmedical use of prescription opioids; VA, Department of Veterans Affairs.

^a^ Results were considered statistically significant when the 95% CIs did not cross 1.

^b^ Diagnoses for opioid, cocaine, and alcohol use disorders were based on *Diagnostic and Statistical Manual of Mental Disorders* (Fourth Edition) criteria.

^c^ Source of opioid was obtained by the 2018 and 2019 National Survey on Drug Use and Health as a self-reported measure.

^d^ Diagnosis for tobacco use disorder was based on nicotine dependence syndrome scale criteria.

**Table 2.** Clinical and Health Care Use Characteristics of US Adults With Pain-Motivated NMUPO Not Using Prescribed Buprenorphine

Person-level characteristic	US adult population^a^ (n = 274 487 145)	Persons with pain-motivated NMUPO not using prescribed buprenorphine^b^ (n = 2359/6 033 424)
Clinical		
Opioid use disorder diagnosis^c^	1 619 474 (0.6)	238/652 213 (10.8)
Methadone prescription in past year	823 461 (0.3)	29/75 418 (1.3)
Source of opioid^d^		
1 Physician	NA	808/2 333 125 (38.7)
>1 Physician	NA	35/98 345 (1.6)
Stole from physician office	NA	17/57 318 (1.0)
From friend or relative for free	NA	958/2 427 850 (40.2)
Bought from friend or relative	NA	169/406 653 (6.7)
Took from friend or relative without permission	NA	76/185 226 (3.1)
Bought from drug dealer	NA	94/204 533 (3.4)
Some other way	NA	110/319 771 (5.3)
No. of medical comorbidities		
0	161 398 441 (58.8)	1547/3 489 129 (57.8)
1-2	103 426 756 (37.7)	763/2 336 745 (38.7)
≥3	9 634 499 (3.5)	49/207 550 (3.4)
Substance use comorbidities		
Cocaine use disorder^c^	1 015 602 (0.4)	62/121 272 (2.0)
Alcohol use disorder^c^	15 645 767 (5.7)	459/1 053 436 (17.5)
Tobacco use disorder^e^	28 793 702 (10.5)	566/1 508 356 (25.0)
Health care use		
No. of ED visits in past year, mean (SD)	0.50 (0.01)	0.94 (0.06)
Any hospital stay in past year	26 570 356 (9.7)	300/850 713 (14.1)
No. of physician visits in past year, mean (SD)	3.4 (0.02)	4.1 (0.14)
At least 1 full agonist opioid taken as prescribed in past year	70 213 812 (26.0)	964/2 582 306 (43.0)

Abbreviations: ED, emergency department; NA, not applicable; NMUPO, nonmedical use of prescription opioids.

^a^ Data are presented as weighted number (weighted percentage) unless otherwise indicated.

^b^ Data are presented as unweighted number/weighted number (weighted percentage) unless otherwise indicated.

^c^ Diagnoses for opioid, cocaine, and alcohol use disorders were based on *Diagnostic and Statistical Manual of Mental Disorders* (Fourth Edition) criteria.

^d^ Source of opioid was obtained by the National Survey on Drug Use and Health as a self-reported measure.

^e^ Diagnosis for tobacco use disorder was based on nicotine dependence syndrome scale criteria.

## Discussion

In this nationally representative sample, pain was a commonly reported motivating factor for NMUPO; however, self-reported use of prescribed buprenorphine was rare despite being likely indicated.^3^ Although analyses were limited by inability to determine clinical severity of NMUPO or indication for buprenorphine (pain vs OUD), we found higher odds of using prescribed buprenorphine associated with characteristics suggestive of higher clinical severity, such as OUD diagnosis or comorbid tobacco use disorder. We also found that the subpopulation not using prescribed buprenorphine reported frequent health care use, and physicians were a common source of misused opioids. Taken together, these findings suggest an opportunity to identify pain-motivated NMUPO at earlier stages of clinical severity through protocolized screening during patient visits to physician offices, emergency departments, and hospitals.

Consistent with prior work,^5,6^ we report lower odds of using prescribed buprenorphine associated with Black race, part-time employment, and no insurance; we extended these findings by demonstrating that disparities persist after controlling for differences in disease prevalence. The marked underuse of and disparities associated with buprenorphine in the setting of pain-motivated NMUPO underscore a need for clinicians treating pain to offer buprenorphine as a core component of services provided.

## Notes

Supplement. **eTable.** NSDUH Questions Used for Cohort and Outcome Variable Definitions
