# Supplementary material for: Self-reported Use of Prescribed Buprenorphine Among US Adults With Nonmedical Use of Prescription Opioids Motivated by Pain
Source: JAMA Netw Open. 2022 Nov 11;5(11):e2241670. doi: 10.1001/jamanetworkopen.2022.41670 (PMC9652749; doi:10.1001/jamanetworkopen.2022.41670)
Supplement: Supplement. — eTable. NSDUH Questions Used for Cohort and Outcome Variable Definitions [file jamanetwopen-e2241670-s001.pdf]

Supplementary Online Content

Buonora MJ, Sung ML, Falker CG, Black AC, Becker WC. Self-reported use of prescribed buprenorphine among US adults with nonmedical use of prescription opioids motivated by pain. *JAMA Netw Open*. 2022;5(11):e2241670. doi:10.1001/jamanetworkopen.2022.41670

eTable. NSDUH Questions Used for Cohort and Outcome Variable Definitions

This supplementary material has been provided by the authors to give readers additional information about their work.

© 2022 Buonora MJ et al. *JAMA Network Open.*

eTable. NSDUH Questions Used for Cohort and Outcome Variable Definitions

DEFINITION COMPONENT	NSDUH variable name	NSDUH question wording	Response required [a]
**COHORT**			
Past-year NMUPO	"pnrnmyr"	"The next question asks about using prescription pain relievers in any way a doctor did not direct you to use them. When you answer these questions, please think only about your use of the drug in any way a doctor did not direct you to use it, including:<br>- Using it without a prescription of your own<br>- Using it in greater amounts, more often, or longer than you were told to take it<br>- Using it in any other way a doctor did not direct you to use it<br>In the past 12 months, did you use [any prescription opioid] in any way a doctor did not direct you to use it?"	Yes
Pain as a motivating factor for use	"pnrrspain"	"Now think about the last time you used a prescription pain reliever in any way a doctor did not direct you to use it… What were the reasons you used [any prescription opioid]? [option 1: To relieve pain?]"	Yes
**OUTCOME**			
Buprenorphine use	"buprpdapyu"	"Please look at the names and pictures of the pain relievers shown below. In the past 12 months, which, if any, of these pain relievers have you used? [options: Suboxone, generic buprenorphine, or other pain relievers that contain buprenorphine]"	Yes
Buprenorphine use without own prescription	"buprpdpymu" + "pnrwynorx"	"In the past 12 months, did you use [buprenorphine] in any way a doctor did not direct you to use it?"<br>+<br>"Which of these statements describe your use of [buprenorphine] in the past 12 months? [option 1: Used pain reliever without own prescription]"	No

[a] The NSDUH survey response required to meet the cohort or outcome definition

© 2022 Buonora MJ et al. *JAMA Network Open.*
